# Hemostasis and Gingival Healing—Polyurethane Adhesive Postextraction Under Rivaroxaban Therapy in a Rodent Model

**DOI:** 10.1155/ijod/3384210

**Published:** 2025-03-13

**Authors:** Marius Heitzer, Philipp Winnand, Marie Sophie Katz, Oliver Grottke, Zuzanna Magnuska, Fabian Kiessling, Frank Hölzle, Ali Modabber

**Affiliations:** ^1^Department of Oral and Cranio-Maxillofacial Surgery, Rheinisch-Westfalische Technische Hochschule Aachen, Aachen, Germany; ^2^Clinic for Anaesthesiology/Operative Intensive Care Medicine, Rheinisch-Westfalische Technische Hochschule Aachen, Aachen, Germany; ^3^Institute for Experimental Molecular Imaging, Rheinisch-Westfalische Technische Hochschule Aachen, Aachen, Germany

**Keywords:** anticoagulants, postextraction bleeding, rivaroxaban, socket healing, tissue adhesives, tooth extraction

## Abstract

**Objectives:** At 31%, the risk of postoperative bleeding after tooth extraction is particularly high in patients who receive rivaroxaban therapy. The aim of this rodent study was to compare the hemostyptic properties and gingival healing between novel polyurethane-based adhesive VIVO and gelatin sponge (GESP) under ongoing rivaroxaban therapy over a period of 10 days.

**Materials:** In total, 120 extractions of the first upper molar were proceeded in rodents treated with rivaroxaban. Of these, 60 postextraction sites were treated with VIVO and 60 with GESP. The duration of the surgical procedure and the clinical parameters of postoperative bleeding and wound evaluation score were recorded. In vivo fluorescence imaging and laser Doppler flowmetry and tissue spectrophotometry (LDF-TS) were performed.

**Results:** GESP provided a faster procedure at 1:06 ± 0:17 min, but postoperative bleeding time was significantly shorter in VIVO sockets at 1:39 ± 0:03 min. Nonsignificant mild bleeding events and comparable wound evaluation scores were recorded in both treatments. LDF-TS showed a significant increase in mean oxygen saturation SO_2_ (%) and mean blood flow (AU) for both treatments. Only GESP showed a significant increase in relative hemoglobin (rHb).

**Conclusion:** In the context of a rodent study, VIVO showed favorable hemostasis and promising gingival healing properties postextraction under ongoing rivaroxaban therapy.

## 1. Introduction

Direct oral anticoagulants (DOACs) are considered the drugs of choice for long-term anticoagulation and are among the most commonly prescribed medications worldwide [1]. Despite the high number of prescriptions, these drugs have considerable disadvantages. Patients who undergo systemic anticoagulation are at particularly high risk of postextraction bleeding [2, 3]. Postextraction bleeding, with a reported probability of up to 31% [4], is, therefore, a particular risk during tooth extractions in patients receiving long-term anticoagulation therapy. Moreover, patients who take anticoagulant and antiplatelet drugs have an increased risk of bleeding as well as an increased risk of wound healing as these drugs modify the hemostatic efficacy and the blood perfusion of the postextraction socket [5]. A change in the blood supply to the gingiva is often accompanied by an inflammatory reaction, which manifests itself through altered oxygen and hemoglobin concentrations and via flow values within the capillary tissue [6–8]. Therefore, additional measurements should be taken intraoperatively in anticoagulated patients to both reduce the risk of postoperative bleeding from the extraction socket and to promote good extraction socket healing [9].

The risk of postoperative bleeding when taking anticoagulants depends on the anticoagulant and the extent of the dentoalveolar surgery. According to the recommendations of the European Heart Rhythm Association (EHRA), patients taking antivitamin K (AVK) drugs can be treated with ongoing anticoagulation with a low risk of postoperative bleeding in the case of low invasive procedures such as tooth extraction of up to three teeth, periodontal surgery, and dental implant placement. For more extensive procedures, these patients should be temporarily bridged with low-molecular-weight heparin [10]. The advantages of the DOACs over AVK include the fact that no INR determination is required, a short half-life and rapid onset of activity, as well as fewer drug-reported drug interactions, which means that bridging therapy does not have to be carried out during oral surgery [10]. EHRA guidelines recommend planning the surgical procedure 18–24 h after the last dose of medication [10]. Furthermore, procedures with a low risk of postoperative hemorrhage can be carried out while taking DOACs without discontinuing the medication.

In recent years, a new hemostatic approach using autologous platelet concentrates to reduce the risk of postoperative bleeding in anticoagulated patients has increasingly come into focus in scientific research [11–13].

Autologous platelet concentrates are blood products used in medical and dental fields to enhance soft and hard tissue healing [13, 14]. In addition to their effective hemostatic properties, autologous platelet concentrates have further wound healing-promoting properties. Due to the presence of growth factors, they support cell proliferation, the production of extracellular matrix, and angiogenesis, among other processes [11]. However, study heterogeneity and potential bias limit direct comparisons between treatment modalities [11, 13]. As a result, the use of platelet plasma has not yet been widely adopted in clinical practice.

In the event of postextraction bleeding, digital compression and sterile gauze strips soaked in saline or tranexamic acid should be applied first. Local hemostatic measures should also be carried out to reduce the risk of postoperative bleeding or to treat the event of postextraction bleeding [11]. In addition to wound sutures of the gingiva, these measures include the use of hemostyptic preparations [15] or the use of blood concentrates [11, 12, 16] within extraction sockets. The hemostyptic preparations used consist of oxidized cellulose, and absorbable gelatin sponges (GESPs) or collagen sponges [15, 17], with absorbable hemostatic sponges in particular being an established local hemostatic agent [15]. However, these preparations pose a risk of allergic, immunological, and even anaphylactic reactions [18, 19]. In addition, the insertion of a collagen sponge into an extraction socket has been demonstrated to be an inferior procedure in terms of wound healing due to inflammatory symptoms, such as redness, swelling, and increased bleeding [15, 20]. Due to the lack of hemostatic effects of these preparations and the unfavorable healing of the extraction sockets in anticoagulated patients, the search continues for alternative procedures to increase the safety of the surgical procedure of tooth extraction under ongoing anticoagulation and to make it more tissue friendly [20, 21].

Substances that improve the healing of oral mucosa wounds are the subject of numerous scientific studies [22, 23]. Tissue adhesives in particular are considered extremely safe for wound care [24–28]. Furthermore, adhesive-based therapy methods are being investigated to reduce the risk of postoperative bleeding after tooth removal and to explore alternatives to GESPs [20, 29, 30]. Most medical tissue adhesives are fibrin based [31]. However, despite their potential hemostyptic effect, the use of fibrin glue after tooth extraction in anticoagulated patients has not been shown to be beneficial [32]. Alternatively, tissue adhesives made from cyanoacrylates have been used successfully in many different procedures and operations [33, 34]. Moreover, studies have shown them to have stronger adhesive strength than fibrin adhesives [33]. In addition, cyanoacrylates have significant limitations based on their low biodegradability, low biocompatibility after polymerization [35], and their lack of approval for clinical use in oral surgery [36]. Beyond having hemostyptic properties, tissue adhesives must also be easy to use in addition to the requirement of favorable interactions with the surrounding tissue with the purpose of good postextraction socket healing with low inflammation [15].

The polyurethane-based tissue adhesive VIVO (Adhesys Medical GmbH, Aachen, Germany) represents an innovative hemostatic tissue adhesive that rapidly and safely polymerizes even in wet tissues [37]. In addition, its favorable degradation of the adhesive components [38] and favorable tissue compatibility [39] over a longer period of time are described in the literature. Due to its favorable biodegradable properties in particular, VIVO appears to be a suitable tissue adhesive for use in extraction sockets. In contrast to other commercially available tissue adhesives, there is relatively little data on the use of VIVO in the oral cavity. In a pilot study conducted in a rodent model, VIVO showed promising hemostyptic results over a short period of time when used in an extraction socket under ongoing anticoagulation [20]. To further evaluate these findings over a longer observation period, the authors hypothesized that the use of the polyurethane-based adhesive VIVO would lead to a reduction in postoperative bleeding and enable good gingival healing after tooth extraction in the context of ongoing DOAC therapy.

## 2. Materials and Methods

All of the experiments were conducted in accordance with the German animal protection law (Tierschutzgesetz, TSchG) and the EU directive (2010/63/EU). The animal protocol was approved by the Governmental Animal Care and Use Committee of the State of North Rhine-Westphalia (Reference No. 81-02.04.2020.A166; Landesamt für Natur, Umwelt und Verbraucherschutz Recklinghausen, Nordrhein-Westfalen, Germany). All of the animals were housed in a pathogen-free environment in filter-top cages (Type 2000, Tecniplast, Buguggiate, Italy) with three rats per cage under a 12-h light/12-h dark cycle. The rats were provided with food and water ad libitum, with soft-soaked food administered. Low-dust wood granulate was used as bedding (Rettenmeier Holding AG, Wilburgstetten, Germany) and as the cage enrichment nesting material (Nestlet, 14010, Plexx B.V., Elst, Netherlands).

A total of 63 adult male Sprague–Dawley rats that were 7 weeks of age and weighed 387.31 ± 21.62 g (Janvier Labs, Le Genest-Saint-Isle, France) were included in this study. In this experimental approach, a male-only animal model was deliberately used, as male animals are not subject to cycle-related hormone fluctuations in comparison to female animals. Particularly considering that female sex hormones influence the structural composition of the gingiva and can therefore have an impact on inflammatory reactions of the gingiva. This manuscript details the investigations and clinical results of this study. According to an established protocol, parenteral administration of rivaroxaban at a therapeutic dose of 3 mg/kg was performed 15 min before the surgical procedure [20]. The administration was repeated daily over a period of 10 days after surgery, and daily injections were administered at the same time each day.

### 2.1. Blood Sample Determination

Rivaroxaban application and determination of blood concentration was performed according to an established protocol [20]. One milliliter of blood was collected from the caudal vein by puncture with a 23G needle after induction of anesthesia with isoflurane (5% by volume), and continuation of inhalation anesthesia with isoflurane (1.5%–2%) and oxygen as carrier gas.

Blood collection was conducted during the experiment under general anesthesia before surgery (T1) and after 10 days (T2), and blood samples were collected in microsample tube sodium citrate (3.2%, Sarstedt). According to a developed protocol standard laboratory methods using the appropriate tests (all from Werfen, Germany) on a ACL-TOP550 (Werfen, Germany) were performed for determination of the rivaroxaban concentrations (HemosIL Liquid Anti-Xa Assay using rivaroxaban calibrators and controls) [20].

### 2.2. Surgical Procedures

**Figure 1:**
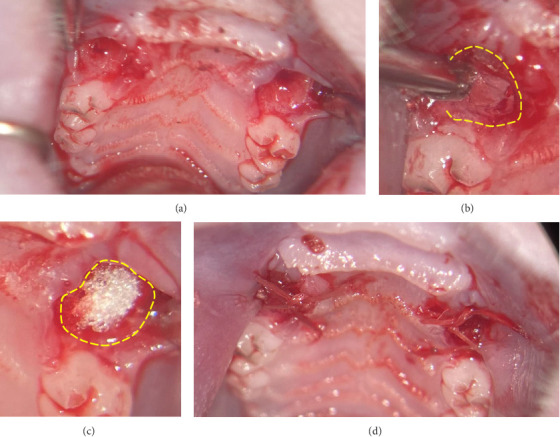
(a) Extraction sockets of first maxillary molar. (b) Application of VIVO and (c) GESP into the extraction sockets. (d) Adapted and sutured gingiva after operation with slight signs of bleeding. GESP, gelatin sponge.

All surgical interventions were performed under ongoing anticoagulation via general anesthesia with intraperitoneal injection of a combination of Medetomidine (0.25 mg/kg) and Ketamine (80 mg/kg) and were performed by one experienced surgeon. The rats were placed in a spine position and a submucosal injection of Ultracaine 4% was administered before the extraction and osteotomy of the first maxillary molars. The osteotomy and extraction procedures were performed under microscopic magnification (OPMI pico f170, Carl Zeiss AG, Oberkochen, Germany). The extraction and osteotomy of the first maxillary molars were performed on both sides. The study was designed as a prospective split-mouth study in rats in which the extraction sockets were hemostyptically treated without randomization as follows: the right extraction socket was treated with VIVO and the left with GESP (ROEKO Gelatamp forte, Coltene, Switzerland). The gingival margins of both groups were then approximated by developing a mucoperistal flap and adapted using single button sutures (Vicryl 6-0, Ethicon Inc., USA) (Figure 1). If there was bleeding, it was stopped by inserting gauze under slight pressure, and the time until the bleeding stopped was recorded as bleeding time. The times of both procedures were recorded from the start of application to the stopping of bleeding after the completion of the sutures. Apart from the surgical procedure and the collection of surgical parameters, all other examinations were carried out by other scientists blinded to the sources.

### 2.3. Bleeding and Healing Examination

After the operation, clinical control of postoperative bleeding was performed three times a day with gentle inspection of the oral cavity. Postoperative bleeding was categorized as mild, moderate, or severe bleeding according to established procedures [2, 17, 20, 40]. The classification of the time of bleeding was based on developed protocols. Immediate postoperative bleeding up to postoperative day 1 was categorized as early bleeding. Postoperative bleeding observed after the second postoperative day was categorized as delayed bleeding [20, 41]. Healing of the extraction socket was assessed according to an introduced Wound Evaluation Scale (WES).

This comprises six added parameters that form a score, which is given a value of 1 if the parameter is absent and 0 if it is not present. A score of six points therefore represents an optimal wound situation. The categories of the score are divided as follows: (1) protruding wound edges, (2) contour irregularities (wrinkling), (3) distance between wound edges >2 mm, (4) edge inversion (sinking, curling), (5) inflammation (redness, discharge), and (6) overall cosmetic appearance (good/not good) [42, 43].

### 2.4. Preparation of Liposomes

The fluorescent Cy7 liposomes were prepared by ethanol dilution. Briefly, 0.246 × 10^−3^ m DPPC (Lipoid, Germany), 0.133 × 10^−3^ m cholesterol (Sigma–Aldrich, Germany), and 0.02 × 10^−3^ m PEG (2000)-DSPE (Lipoid, Germany) were diluted in 10 mL chloroform (AppliChem, Germany) together with 0.75 × 10^−9^ m Cy7-DPSE (Avanti Polar Lipids, United States). Under a low vacuum at 70°C, the solution was dried for 1 h in the rotary evaporator. Then, 10 mL PBS was added and stirred without vacuum at 70°C. To extrude, liposomes were filtered twice at 200 nm, three times 100 nm (Liposofast LF-50; Avestin, Germany), and sterilized with a 0.2 µm syringe filter. Characterization was performed with DLS (Zetasizer Nano; Malvern, UK) and a plate reader (M200pro; Tecan, Switzerland) for fluorescence with excitation set at 745 nm and emission set 775 nm [44]. The synthesized Cy7 liposomes have a diameter of 114 nm, a polydispersity index (PDI) of 0.07, and a zeta potential of −9.33 mV based on DLS measurements.

### 2.5. In Vivo Fluorescence Imaging

The in vivo near infrared (NIR) fluorescent imaging was performed during anesthesia using a μCT system (U-CT OI, MILabs, Utrecht, the Netherlands) immediately postoperative (T1) and after 10 days (T2). The µCT scans were acquired with ultrafocus magnification by 360° rotation at 0.75° increments with 0.3 s/°. Intravenous application of the Cy7-labeled liposomes was conducted 1-min prior to imaging. Fluorescence imaging was performed first. The animal was placed in a holder that automatically moved to the front of the imaging device—between the laser and the cooled CCD camera. A laser and filter with excitation and emission wavelengths of 710 and 775 nm were used to generate excitation and emission images. The recorded data were reconstructed as 2D images. After acquisition of the fluorescence scan, the animal holder was automatically moved to the μCT unit to acquire a CT scan of the animal's jaw. µCT imaging was performed at a voltage of 65 kV, a current of 0.13 mA, and an exposure time of 300 ms. The scans were acquired using ultra-focus magnification through 360° rotation in 0.75° increments at 0.3 s/°, and the data were reconstructed with an isotropic voxel size of 40 µm. The CT scans were matched with the 2D fluorescence images. The 2D images obtained with NIR imaging were analyzed for total photon count (TPC) and mean fluorescence intensity (MFI). To extract these parameters, a fluorescent signal in the oral cavity of the rat was segmented within a circular region of interest (ROI) [45]. The obtained segments were divided into left (GESP) and right (VIVO). This operation was reproduced for each subject and for both time points.

### 2.6. In Vivo Detection of Gingival Capillary Parameters

In vivo measurements and examinations were performed to investigate the gingival blood flow and thus enable quantification and graduation of the inflammation severity of the gingival healing after the dental extraction. All of the measurements started with the use of the laser Doppler flowmetry and tissue spectrophotometry (LDF-TS), and all rodents were measured and examined by the same experienced physician. Gingival perfusion was analyzed with the LDF-TS “oxygen to see” (O_2_C) device (LEA-Medizintechnik, Gießen, Germany). Short, noninvasive measurements were performed at T1 and T2.

The O_2_C device is an established device in the clinical noninvasive monitoring of microsurgical flaps and enables the generation of quantified perfusion data in the form of oxygen saturation SO_2_ (%), relative hemoglobin (rHb) arbitrary units (AUs), and blood flow (AU) [46, 47]. Depending on what probe is used, intraoral use is also possible to measure blood flow and discriminate between healthy and inflamed gingiva [6]. All measurements used the LFX-19 probe (LEA-Medizintechnik, Gießen, Germany). The probe head in our study was specifically manufactured for intracorporal measurements in animals and has a predefined measurement depth of 1 mm. To measure the superficial oral rat mucosa, a specially made glass spacer plate with a thickness of 0.6 mm was attached to the probe. Two measurements were conducted per extraction socket.

### 2.7. Statistical Analysis

Sample size calculation was performed software-based using G^*⁣*^*∗*^^Power (G^*⁣*^*∗*^^Power, Version 3.1.9, Düsseldorf, Germany). The a-priori Wilcoxon–Mann–Whitney test was used for the two groups. The significance level of 0.05 and the effect size of 0.62 were chosen based on the reported risk of postextraction bleeding when hemostatic agents are used in patients undergoing antithrombotic therapy [48]. Against this background and power of 95%, at least 60 extraction sockets per group were defined as necessary parameters.

Data analysis was conducted using GraphPad Prism 7.0 (GraphPad Software, Inc., La Jolla, San Diego, CA, USA). The Mann–Whitney U test was used for nonparametric independent variables to compare the differences between the parameters of operation time, bleeding time, and WES. The Kruskal–Wallis test was used for nonparametric analysis of the NIR parameter and the LDF-TS parameter. All data represented the means ± standard deviation (SD), and statistical significance was assessed at a level of *p* ≤ 0.05.

## 3. Results

### 3.1. Blood Sample Determination

**Figure 2:**
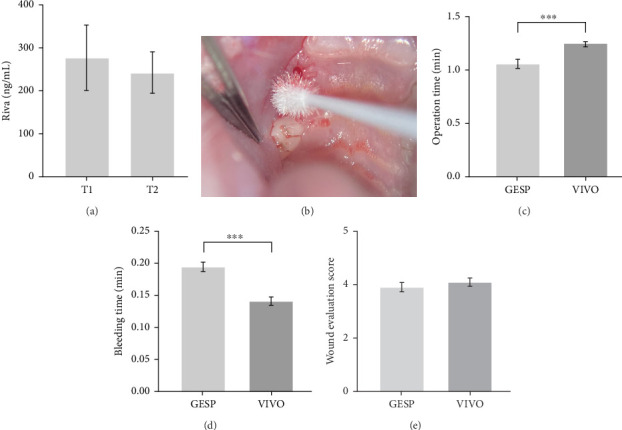
(a) Graphical representation of the blood analysis. Rivaroxaban concentrations at blood drawing before operation (T1) and after 10 days (T2). (b) Picture of postoperative bleeding and compression on extraction socket. (c) Graphical representation of the operation time, (d) bleeding time, and (e) wound evaluation score; *⁣*^*∗∗∗*^*p* ≤ 0.001.

This study comprises the evaluation of a total of 120 extraction sockets of a split-mouth model under continuous anticoagulation of 60 rats with rivaroxaban. At the time of oral surgery T1, the rivaroxaban concentration determined from the venous blood was 276.9 ± 273.3 (ng/mL). After 10 days, at time T2, the rivaroxaban concentration was 241.6 ± 184.2 (ng/mL) (Figure 2).

### 3.2. Surgical Procedures

**Figure 3:**
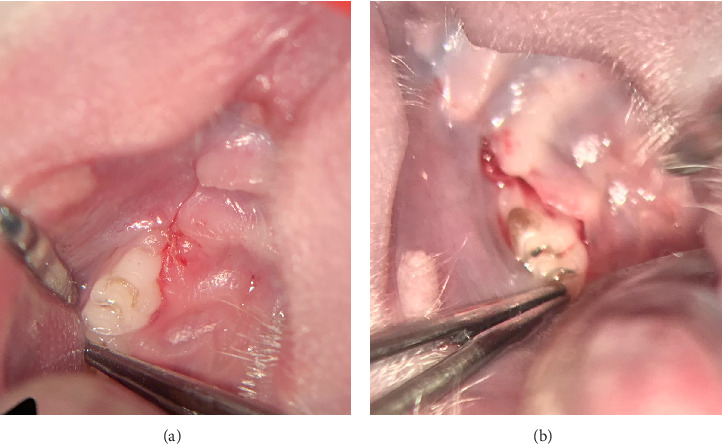
Figures of exemplary extraction sockets treated with VIVO were assessed using the wound evaluation score. Panel (a) shows an extraction socket with good wound healing, slight redness indicating mild inflammation, and remnants of dissolvable suture material, rated a total of five points. (b) This example shows an extraction socket rated three points due to contour irregularities, signs of inflammation, and reduced overall esthetics. The wound edges are less than 2 mm apart, so this is not a contributing factor.

Of the total of 120 extraction sockets, hemostyptic therapy was performed with GESP in 60 extraction sockets and with VIVO in 60 extraction sockets. The total time required for the entire procedure (hemostatic application and hemostasis achievement) using GESP was significantly shorter at 1:06 ± 0 : 17 (min) compared to the VIVO procedure with 1:24 ± 0:07 (min) (*p* ≤ 0.001). In contrast, the postoperative bleeding time in the extraction sockets treated with VIVO was significantly shorter at 0.14 ± 0.03 (min) compared to the GESP group at 0.19 ± 0.02 (min) (*p* ≤ 0.001) (Table 1, Figures 2 and 3).

### 3.3. Bleeding and Healing Examination

Oral examinations showed no significant differences between the two treatment forms of GESP and VIVO in terms of degree of bleeding. Thirteen mild bleeding events (21.67%) with 0.22 ± 0.42 were observed in the extraction sockets with GESP and 11 mild bleeding events (18.33%) with 0.18 ± 0.39 in the extraction sockets with VIVO (*p*=0.82). No moderate or severe postextraction bleeding was detected in either group. There were also no differences in the incidence of postextraction bleeding between the two treatment forms. Four early bleedings occurred in the GESP group and one in the VIVO group (*p*=0.36). The number of delayed bleeding events was nine in the GESP-treated extraction sockets and 10 in the VIVO-treated extraction sockets (*p*=0.99). GESP was associated with a higher absolute postoperative bleeding risk of 21.67% compared to VIVO at 18.33% (Table 2). After 10 days, there was no significant difference in the wound evaluation score of the extraction sockets treated with GESP, with scores of 3.94 ± 0.75 as compared to the VIVO group, which had scores of 4.12 ± 0.58 (Figure 2).

### 3.4. In Vivo Fluorescence Imaging

**Figure 4:**
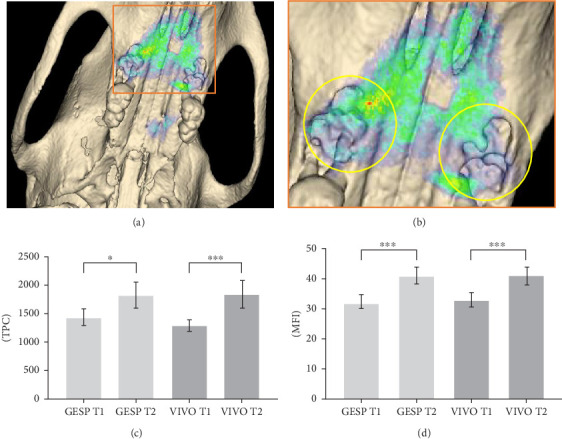
Graphical representation of 2D images of NIR. (a) 2D overview image and (b) enlarged view with marking of the ROIs. NIR evaluation of (c) TPC and (d) MFI of VIVO and GESP at T1 and T2. *⁣*^*∗*^*p*=0.017, *⁣*^*∗∗∗*^*p* ≤ 0.001. GESP, gelatin sponge; MFI, mean fluorescence intensity; NIR, near infrared; ROIs, region of interest; TPC, total photon count.

Noninvasive NIR imaging revealed an increase in TPC and MFI during the 10-day observation period. The soft tissue located in the ROI of the extraction sockets treated with GESP increased from 1425 ± 412.2 TPC at T1 to 1832 ± 713.8 TPC (*p*=0.017) at T2. Those treated with VIVO increased from 1283 ± 306.8 TPC at T1 to 1850 ± 745.1 TPC (*p* ≤ 0.001) at T2. The MFI significantly increased for both the GESP and VIVO treatments (*p* ≤ 0.001) at day 10 (T2). There was no difference in TPC and MFI values between GESP and VIVO treatments (Figure 4).

### 3.5. In Vivo Measurement of Gingival Capillary Parameters

**Figure 5:**
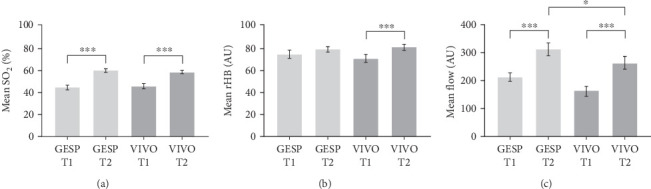
Graphical representation of LDF-TS analysis. (a) Mean oxygen saturation, (b) the relative amount of hemoglobin, and (c) flow values compared between gingiva of VIVO and GESP at T1 and T2. *⁣*^*∗*^*p*=0.02, *⁣*^*∗∗∗*^*p* ≤ 0.001. GESP, gelatin sponge; LDF, laser Doppler flowmetry; TS, tissue spectrophotometry.

Noninvasive in vivo measurement by LDF-TS at T1 and T2 showed that mean oxygen saturation SO_2_ (%) and mean blood flow (AU) increased significantly under both treatment conditions (both *p* ≤ 0.001), while an increase in mean rHb (AU) was only observed in the VIVO-treated extraction sockets (*p* ≤ 0.001). No differences in mean SO_2_ (%) or mean rHb (AU) were found between the two groups at either time point. The mean blood flow at 315.2 ± 133.0 AU was significantly increased in the GESP postextraction group compared to the VIVO-treated group at 275.5 ± 126.8 AU at T2 (*p*=0.02) (Figure 5).

## 4. Discussion

This manuscript compares the evaluation of hemostyptic effect and impact on soft tissue wound healing postextraction between the novel polyurethane-based adhesive and the widely used GESP within a rivaroxaban–anticoagulated rodent model over an observation period of 10 days. The increasing number of patients taking DOACs (1), poses an increasing challenge for oral surgeons treating this patient group [1, 9, 36, 49]. With around 76%, rivaroxaban is the most commonly used medication by patients undergoing dental surgery [50]. Although a reduction in the risk of postextraction bleeding by modifying drug anticoagulation has been widely discussed in the literature [1, 9, 36, 49–51], interruption of oral anticoagulation during these procedures is considered unfavorable as it may be associated with an increased risk of embolic events [10, 52, 53]. Therefore, minor oral surgery can be performed while using DOACs [9, 10, 17, 51].

This study investigated and compared the effects on hemostasis of inserting a GESP versus the intra-alveolar application of a polyurethane adhesive in patients undergoing therapeutic anticoagulation with rivaroxaban. GESPs are frequently used in oral surgery due to their hemostyptic, biocompatible, and biodegradable properties [54]. Anticoagulation and the associated compromise of blood coagulation were performed in a rodent model by intravenous administration of rivaroxaban. One limitation of our study was the intravenous administration of an oral anticoagulant drug; however, rivaroxaban is subject to different pharmacological dynamics depending on the route of administration. On the other hand, intravenous administration of rivaroxaban within an animal model at a dose of 3 mg/kg body weight is a more reliable and safer compared to oral administration of anticoagulants [20, 55] as it is not subject to the variability of oral administration due to the animals' food intake or the life-threatening intratracheal misapplications due to administration by gavage [20]. In an attempt to conduct an animal-friendly and well-established procedure for investigations on postextraction bleeding, the authors opted for the intravenous form of administration of rivaroxaban, as described in this manuscript.

Therapeutic anticoagulation with 3 mg/kg rivaroxaban in a rodent model is described in the literature with venous concentrations of 203.53–387.7 ng/mL of rivaroxaban [20, 56]. According to the concentrations described in the literature, the venous rivaroxaban concentrations determined for use in this study were 241.6–276.9 ng/mL, which demonstrates that therapeutic anticoagulation was present as a prerequisite for the evaluation of hemostatic material properties in our study.

The operation time of 1:06 ± 0:17 min in the GESP group was significantly faster than the operation time of 1:24 ± 0:07 min in the VIVO group, although the authors are of the opinion that the difference of 18 s is almost negligible in a tooth extraction procedure with hemostatic measures. However, the effectiveness of the hemostatic agent is clinically crucial in this context [15, 17]. A quality criterion for the effectiveness of a hemostatic agent is the time of bleeding after extraction, which can be divided into primary prolonged postoperative bleeding, early bleeding, and delayed bleeding [2, 17, 20, 40]. Primary prolonged postoperative bleeding events were observed in both groups, but the postoperative bleeding time in the extraction sockets treated with VIVO was significantly shorter compared to the GESP treatment, at 1:39 ± 0:03 min and 1:55 ± 0.02 min, respectively. No difference was observed between the two forms of therapy in terms of the number of early and delayed bleedings.

In addition, Abdullah and Khalil [40] categorized the quality of bleeding after extraction as mild, moderate, or severe in a study on risk stratification of tooth extractions under ongoing anticoagulation. In our study, 13 mild postextraction bleedings occurred in the sockets treated with GESP, and 11 postextraction bleedings occurred in the sockets treated with VIVO, with no significant difference. Neither moderate nor severe bleeding was observed after extraction in either group with anticoagulation. In the literature, the risk of postextraction bleeding in anticoagulated patients is stated as being up to 31% [4]. Although both procedures showed lower postoperative bleeding risks compared to the literature, the use of GESP was associated with a higher absolute postoperative bleeding risk of 21.67%, compared to 18.33% for VIVO. In addition, the use of VIVO showed better hemostyptic properties in terms of a shorter bleeding time immediately postoperative.

Platelet concentrates, such as platelet-rich plasma (PRP) [16] and autologous leukocyte- and platelet-rich fibrin (L-PRF) gel [12, 14], have shown promising results in extraction sockets, helping prevent postoperative bleeding without altering oral anticoagulant therapy [12]. They also aid in managing postoperative pain and promoting soft tissue healing [13, 14]. Analogous to the descriptions in the literature regarding the use of platelet concentrates, a good healing of the soft tissue could be observed after 10 days of using VIVO. On the one hand, autologous platelet plasma products have the significant advantage of being derived from the same individual, allowing for use with minimal immunological interactions. However, the fact that autologous platelet plasma products require extensive processing before application leads to limitations such as long preparation times and high costs [16]. The short curing time of the polyurethane adhesive used represents a considerable time benefit compared to the use of blood products. Another limitation for the use of platelet concentrates is the inconsistent study data. Although the data in the individual studies are very promising, the heterogeneous study designs currently pose significant limitations for comparability with the use of common hemostatic agents for postoperative bleeding prophylaxis in anticoagulated patients [11, 13].

Apart from a reduction of postoperative bleeding, another important requirement for a hemostyptic preparation is whether it has favorable properties for soft tissue wound healing of the gingiva [14, 20]. This is particularly important for patients on medication that affects blood coagulation, who are at increased risk of delayed and compromised wound healing [5]. Although the literature describes inferior wound healing under anticoagulation when using a collagen sponge compared to procedures with adhesive agents for the prophylaxis of postextraction bleeding due to increased inflammatory symptoms such as redness, swelling, and increased bleeding [15, 20], the wound evaluation score used in this study showed no difference between the two types of therapy. A major limitation of the study is the lack of accurate information over a longer period of time, as wound assessment was performed after a longer observation period [15]. As wound healing is dependent on many factors, such as wound size and time [17], the wound assessment between these studies must be viewed critically because an equal observation period is obligatory for a wound comparison. Yet another limitation of wound evaluation scores in general is that wound assessment is an inherently subjective process.

The blood perfusion and the blood flow of the postextraction socket have significant influences on wound healing and can be altered by antihemostatic drugs [5]. In vivo fluorescence imaging with cyanin dyes in small animals has emerged in recent years and is now considered a promising technique for tissue examination [39, 57, 58]. In contrast to the frequently used indocyanine green, novel peptide-based NIR fluorescent molecular probes are associated with the avoidance of dye leaching and longer fluorescence lifetimes [57]. The properties of Cy7-labeled liposomes are such that they strongly accumulate by enhanced permeability and retention (EPR) effect in tumor tissue, inflammatory tissue, or regenerating tissue which enables noninvasive living imaging of tissues [44, 59]. After 10 days, physiologically advanced wound healing and angionesis of soft tissue are associated with an increase in the fluorescence signal of intravenous fluorescent dyes [39]. The EPR effect is based on the accumulation of Cy7 when the one hand, vascular permeability is increased and, on the other, venous drainage is impaired [59]. Adhesives have the potential to positively influence angiogenesis during the wound healing process [28]. Accordingly, a significant increase in the fluorescence signal was observed after 10 days in both of the study groups, whereby the significant increase in the NIR in relation to the TPC was more pronounced in the gingival tissue of the VIVO-treated group (*p* ≤ 0.001).

LDF-TS in vivo measurements offer the advantage of enabling objective assessment and quantification of blood flow. They also represent an evidence-based tool for differentiating between healthy or inflamed gingiva [6]. The O_2_C device is used to measure postcapillary oxygen saturation and hemoglobin concentration as well as blood flow at local points [6]. In our study, the mean SO_2_ value measured by LDF-TS was significantly higher at T2 compared to T1 for both the GESP and VIVO groups, with no significant difference between the two treatments. In the literature, a higher percentage of oxygen has been demonstrated to be a sign of lower oxygen consumption and may indicate the hypoxic conditions of an inflammatory tissue [6–8]. In this context, a high hemoglobin concentration is described as a warning sign of degenerative vasoconstriction [6–8], which was observed between T1 and T2 within the VIVO group. The clinical symptoms of an inflamed gingiva, such as redness and swelling, indicate a state of hyperemia and are associated with increased flow values [6]. In our study, significantly increased flow values were detected between T1 and T2. Relative flow values of 315.2 AU in the tissue of the GESP extraction socket were significantly increased when measured after 10 days in addition to the relative flow values of the VIVO group at 267.5 AU. These measurements were analogous to the tendency of the GESP group's wound healing evaluation score to be worse after 10 days, making VIVO a treatment procedure that was associated with fewer capillary changes and less inflamed gingiva in the course of wound healing after 10 days.

The strengths of the presented animal study are related to the testing of the hemostyptic effect of GESP and VIVO in the form of a split-mouth design under standardized and comparable conditions. On the other side, the main limitation of this exploratory rodent study is the fact that the authors reduced the number of experimental animals in accordance with the 3R rules and did not use a control group without local hemostyptic measurements. Moreover, small animal models cannot completely reproduce the anatomical situation of human patients. In addition, rats do not have the same level of blood coagulation as humans, which may influence the evaluation of postextraction hemorrhage.

## 5. Conclusion

Within the limitations of an extraction model in anticoagulated rodents, the use of a novel polyurethane-based adhesive showed promising results over an observation period of 10 days with regard to its hemostyptic effects under ongoing therapy with DOACs. The tissue adhesive VIVO was associated with a lower absolute risk of postoperative bleeding and showed lower inflammatory capillary parameters of the gingival tissue after a healing phase of 10 days compared to the treatment of GESP.

## Figures and Tables

**Table 1:** All data represented the means ± standard deviation (SD).

Parameter	Rats (*n* = 60)
Weight (g)	387.31 ± 21.62
Operation time (min)	
GESP	1.06 ± 0.17
VIVO	1.24 ± 0.07
Bleeding time (min)	
GESP	0.19 ± 0.02
VIVO	0.14 ± 0.03
Wound evaluation score	
GESP	3.93 ± 0.75
VIVO	4.12 ± 0.58

**Table 2:** Degree, timing, and absolute probability of bleeding.

Group	Degree of bleeding mean ± SD						Early bleeding mean ± SD		Delayed bleeding mean ± SD		Absolute postextraction bleeding probability (%)
	Mild		Moderate		Severe						
GESP (*n* = 60)	13/60 (*p*=0.82)	0.22 ± 0.42	0/60	0.00	0/60	0.00	4/60 (*p*=0.36)	0.07 ± 0.25	9/60 (*p* > 0.99)	0.15 ± 0.36	21.67
VIVO (*n* = 60)	11/60 (*p*=0.82)	0.18 ± 0.39	0/60	0.00	0/60	0.00	1/60 (*p*=0.36)	0.02 ± 0.13	10/60 (*p* > 0.99)	0.17 ± 0.38	18.33

*Note:* All data represented the means ± standard deviation (SD). VIVO, polyurethane adhesive.

Abbreviation: GESP, gelatin sponge.

## Data Availability

The data presented in this study are available upon request from the corresponding author.
